# Cost-effectiveness of first-line enfortumab vedotin in addition to pembrolizumab for metastatic urothelial carcinoma in the United States

**DOI:** 10.3389/fimmu.2024.1464092

**Published:** 2024-09-09

**Authors:** Andong Li, Meiyu Wu, Ouyang Xie, Heng Xiang, Kehui Meng, Chongqing Tan, Long Wang, Xiaomin Wan

**Affiliations:** ^1^ Department of Pharmacy, The Second Xiangya Hospital, Central South University, Changsha, Hunan, China; ^2^ Institute of Clinical Pharmacy, Central South University, Changsha, Hunan, China; ^3^ Department of Urology, The Third Xiangya Hospital, Central South University, Changsha, Hunan, China

**Keywords:** enfortumab vedotin, pembrolizumab, bladder cancer, cost-effectiveness, the United States

## Abstract

**Background and objective:**

The EV-302 trial found that the combination of enfortumab vedotin (EV) with pembrolizumab significantly improved survival for patients with metastatic urothelial carcinoma (mUC). However, given the high cost of the drugs, there is a need to assess its value by considering both efficacy and cost. This study assessed the cost-effectiveness of EV plus pembrolizumab as a first-line treatment for patients with mUC from the perspective of U.S. payers.

**Methods:**

A Markov model was developed to compare the lifetime costs and effectiveness of EV in combination with pembrolizumab with chemotherapy in the treatment of mUC patients from U.S. payer perspective. Life-years (LYs), quality-adjusted LYs (QALYs), and lifetime costs were estimated. One-way, two-way and probabilistic sensitivity analyses were conducted to evaluate model uncertainty. Additionally, subgroup analyses were performed.

**Results:**

Compared to chemotherapy, the combination of EV and pembrolizumab provided an additional 2.10 LYs and 1.72 QALYs, at an incremental cost of $962,240.8 per patient. The incremental cost-effectiveness ratio (ICER) is $558,973 per QALY. Subgroup analysis indicated that patients ineligible for cisplatin treatment had a lower ICER compared to those who were eligible for cisplatin.

**Conclusions:**

From the perspective of US payers, at a willingness-to-pay threshold of $150,000 per QALY, the combination of EV and pembrolizumab is estimated to not be cost-effective compared to traditional chemotherapy in the first-line treatment of mUC patients.

## Introduction

Bladder cancer is one of the ten most commonly diagnosed cancers in the United States, accounting for 4.2% of all new cancer cases in 2023 (1). About 90-95% of bladder cancer cases are urothelial carcinoma (2, 3). Patients with metastatic urothelial carcinoma(mUC) have a poor prognosis, with a five-year survival rate of only 5-7% (1).

Platinum-based chemotherapy is the standard of care for previously untreated patients with mUC (4), however, the clinical outcomes associated with this regimen remain suboptimal (5). Immunotherapy has become increasingly popular in the field of cancer treatment due to its remarkable efficacy, as seen in the treatment of breast and thyroid cancers (6, 7). PD-1 and PD-L1 inhibitors are commonly used in patients who are ineligible for Platinum-based chemotherapy, as a follow-up therapy after platinum-based chemotherapy, or as an alternative treatment for recurrent or resistant cases (8). Despite the use of these inhibitors in mUC, many patients still experience progression (9). Enfortumab Vedotin (EV), an antibody-drug conjugate directed against nectin-4 (10), received breakthrough therapy designation from the U.S. Food and Drug Administration (FDA) in 2018 (11), followed by marketing approval in December 2019 for its use as a second-line treatment in patients with mUC (12). It is indicated for patients with locally advanced or metastatic bladder cancer who have previously received platinum-based chemotherapy and immune checkpoint inhibitor (ICI) therapy, and its use for second-line treatment of mUC is recommended by NCCN guidelines (8).The EV-302 trial evaluated the combination of EV and pembrolizumab for previously untreated patients with mUC (13). This pivotal phase 3 trial demonstrated a significant survival benefit for patients receiving EV plus pembrolizumab compared to chemotherapy, reducing the risk of death by 53% and nearly doubling median overall survival (OS), with hazard ratio (HR) of 0.47 (median OS: 31.5 months vs. 16.9 months). The combination therapy also reduced the risk of progression or death by 55% and nearly doubled median PFS, with an HR of 0.45 (median PFS: 12.5 months vs. 6.3 months). Based on these results, the FDA has granted approval for EV plus pembrolizumab as a first-line treatment for patients with mUC (14). Although the trial demonstrated a near doubling of both median OS and PFS, it remains unclear from a value perspective whether the cost of this therapy is justified by its potential benefit. The aim of this study was to evaluate the cost-effectiveness of EV combined with pembrolizumab versus platinum-based chemotherapy as first-line treatment in patients with mUC from the perspective of U.S. payers.

## Material and methods

**Figure 1:**
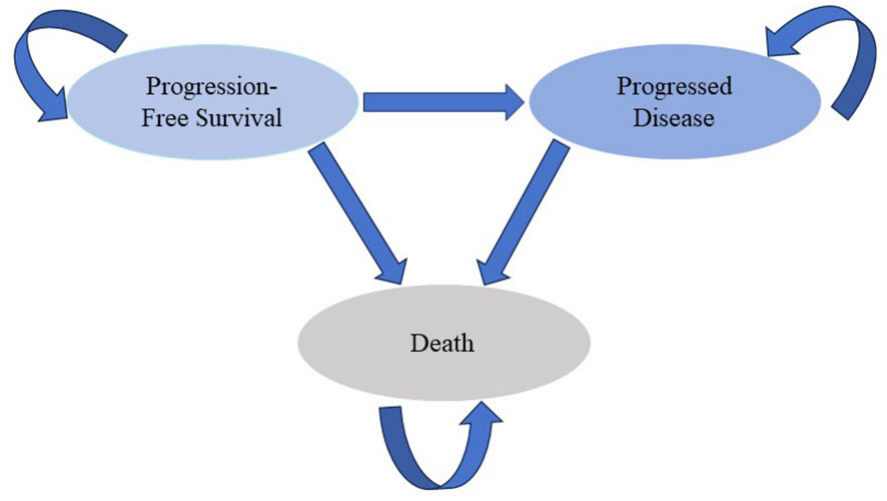
Markov Model. PFS, progression-free survival; OS, overall survival.

A Markov model was developed to estimate the costs and effectiveness of the first-line treatment for patients with mUC (**Figure 1**) (15, 16). Two first-line treatment options were evaluated in the model: 1) the combination of EV with pembrolizumab, and 2) chemotherapy consisting of cisplatin or carboplatin plus gemcitabine. We assumed that first-line treatment would continue until disease progression, at which point both groups could receive second-line treatment until death.

The model considered only direct medical costs, with costs and outcomes discounted annually at a rate of 3% (17). Each model cycle represented 3 weeks. The time duration was lifetime. Half-cycle correction was applied in the model. The outputs included total costs, life-years (LYs), quality-adjusted life-years (QALYs), and incremental cost-effectiveness ratios (ICERs). To determine the cost-effectiveness of therapy, a willingness-to-pay (WTP) threshold of $150,000 per QALY was used, as recommended by Neumann et al (18). The development of the Markov model and statistical analyses were performed using R 4.2.3 software (http://www.r-project.org).

### Model progression and survival estimates

The risks of disease progression and overall mortality in each treatment group were evaluated based on the PFS and OS curves of the EV-302 study (13). PFS and OS probabilities were extracted utilizing the WebPlotDigitizerwebsite (https://apps.automeris.io/wpd/index.zh_CN.html). Subsequently, pseudo-individual patient data were generated using the methodology proposed by Guyot et al (19). These data were then fitted with various parametric distributions, including exponential, weibull, log-logistic, log-normal, gompertz, generalized gamma, spline and mix cure distributions. Based on the goodness-of-fit evaluation using the Akaike Information Criterion (AIC), a log-normal distribution was selected for the OS curve, and a spline distribution was chosen for the PFS curve in the chemotherapy arm (**Supplementary Table 1**). The hazard rates for the EV plus pembrolizumab arm were estimated by multiplying the hazard rates for the chemotherapy arm by the corresponding HRs. The background mortality rate for each age group was estimated based on US life tables (20).

### Cost and utility estimates

Direct medical costs included the cost of the drug, administration, best supportive care, maintenance therapy and management of adverse events (AEs). In the EV group, pembrolizumab was administered at a dose of 200 mg on the first day of each cycle, for a maximum of 35 cycles, while EV was administered at a dose of 1.25 mg/kg on the first and eighth days of each cycle, with no maximum treatment duration (13). After disease progression, it was assumed that all patients receive chemotherapy as second-line treatment based on the NCCN guidelines (8). In the chemotherapy group, gemcitabine was dosed at 1000 mg per square meter of body surface area (BSA) and administered via intravenous injection on the first and eighth days of each model cycle. Carboplatin (administered intravenously with an area under the curve of 4.5 mg/mL/min) or cisplatin (administered intravenously at 70 mg/m2 BSA) was given on the first day of each cycle (13). In the chemotherapy group, after reaching the maximum treatment cycles of chemotherapy, 30.2% of patients used avelumab as maintenance therapy based on the EV-302 trial (13). Similarly, it was assumed that after progression, patients in the chemotherapy arm receive pembrolizumab according to the NCCN guidelines (8). The drug costs were based on the average sale price from the Centers for Medicare and Medicaid Services for the year 2023 and published studies (9, 21). The costs of adverse events were derived from previously published studies (9, 22–24). Administration costs were estimated according to the Medicare physician fee schedule for the year 2023 (25). All information regarding costs is listed in **Table 1**. The model used a body weight of 70 kg, a body surface area of 1.86 m2, and a creatinine clearance rate of 70 mL/min for dose calculations, as sourced from published literature (26). The impact of Grade 3 or 4 AEs was considered in the model as measured by health disutility weight and the cost of the AEs (**Table 1**) (27–30). All costs were converted to 2023 US dollars using the Consumer Price Index for medical care (31).

**Table 1:** Model parameters: baseline values, ranges, and distributions for sensitivity analysis.

Parameter	Baseline value	Range	Distribution	References
Chem AEs incidence				
Anemia	0.314	0.251 - 0.377	Beta	13
Neutropenia	0.300	0.240 - 0.360	Beta	13
Thrombocytopenia	0.194	0.156 - 0.233	Beta	13
Decreased neutrophil count	0.090	0.072 - 0.108	Beta	13
EV+pemb AEs incidence				
Neutropenia	0.048	0.038 - 0.058	Beta	13
Maculopapular rash	0.077	0.062 - 0.092	Beta	13
Hyperglycemia	0.050	0.040 - 0.060	Beta	13
Utility				
PFS	0.800	0.770 – 0.820	Beta	32
PD	0.750	0.700 – 0.790	Beta	32
AEs disutility				
Anemia	0.120	0.096 - 0.144	Beta	28
Decreased neutrophil count	0.090	0.072 - 0.108	Beta	29
Hyperglycemia	0.140	0.112 - 0.168	Beta	30
Neutropenia	0.150	0.120 - 0.180	Beta	28
Maculopapular rash	0.032	0.026- 0.039	Beta	27
Thrombocytopenia	0.110	0.088 - 0.132	Beta	28
AEs cost, $				
Anemia	4,638.000	3,710.4 - 5,565.6	Gamma	9
Decreased neutrophil count	36,106.000	28,884.800 - 43,327.200	Gamma	23
Hyperglycemia	255.506	204.405 - 306.607	Gamma	24
Neutropenia	17,181.000	13,744.800 - 2,0617.200	Gamma	9
Maculopapular rash	15,709.000	12,567.200 - 18,850.800	Gamma	9
Thrombocytopenia	45,332.000	36,265.600 - 54,398.400	Gamma	22
Patients’ weight, kg	70.000	60.000 - 140.000	Gamma	26
Drug cost per milligram, $				
Enfortumab Vedotin	131.520	116.36 - 174.54	Gamma	9
Pembrolizumab	55.730	46.893 - 70.339	Gamma	21
Carboplatin	0.072	0.058 - 0.086	Gamma	21
Cisplatin	0.404	0.323 - 0.485	Gamma	21
Gemcitabine	0.044	0.035 - 0.053	Gamma	21
Avelumab	9.236	7.389 - 11.083	Gamma	21
Administration cost				
First hour	144.390	115.512 - 173.268	Gamma	25
Additional hour	31.100	24.880 - 37.320	Gamma	25
Best support care/cycle,$	1,213	970.4 – 1455.6	Gamma	9
Creatinine clearance	70			26
Body surface area (m^2^)	1.86	1.456-2.184	Normal	26
HR of PFS	0.47	0.38 - 0.58	Log-normal	13
HR of OS	0.45	0.38 - 0.54	Log-normal	13

Chem, chemotherapy; pemb, pembrolizumab; AE, adverse event; EV, enfortumab vedotin; PFS, progression-free survival; OS, overall survival; HR, hazard ratio.

Health utility values for all health states were derived from published studies. Utilities of 0.80 and 0.75 were assigned to patients receiving first-line and second-line therapy, respectively (32). The loss of QALYs due to AEs was estimated by multiplying the incidence rates of the AEs by their corresponding disutility values.

### Sensitivity analysis

A series of sensitivity analyses were conducted to assess the uncertainty of parameters and identify which parameters had the greatest impact on the results. In probabilistic sensitivity analysis, 1,000 Monte Carlo simulations were performed with the parameters simultaneously varied with a specific pattern of distribution (**Table 1**). In one-way sensitivity analysis, each parameter is independently and singly varied within ±20% of the baseline value or within its 95% confidence interval to assess the impact on the model results. A two-way sensitivity analysis was conducted to evaluate the interaction between the utility values for EV combined with pembrolizumab and platinum-based chemotherapy. In this analysis, the utility values for both treatment arms were simultaneously varied, ranging from -50% of the baseline value up to 1. Additionally, we conducted scenario analyses assuming different unit prices for EV and pembrolizumab.

The EV-302 trial presented survival curves for multiple biomarker subgroups (13). To assess the cost-effectiveness of EV in combination with pembrolizumab across different patient subgroups, the ICER was estimated for each subgroup using the PFS and OS curves, following the same methodology as in the baseline analysis.

## Results

### Base case results

Based on the model projection, patients receiving combination therapy with EV and pembrolizumab had an estimated life expectancy of 4.221 LYs, representing a gain of 2.10 LYs compared to those receiving chemotherapy. When accounting for quality of life, patients on the EV and pembrolizumab gained 3.254 QALYs, an improvement of 1.721 QALYs compared to patients on chemotherapy. The combination regimen incurred an additional cost of $962,240.8 per patient compared to chemotherapy. As a result, the ICER for EV plus pembrolizumab compared to chemotherapy was $558,973 per QALY ($458,390.1 per LY) (**Table 2**).

**Table 2:** Base case results.

Results	EV+Pembrolizumab	Chemotherapy	Incremental
LYs	4.221	2.121	2.100
QALYs	3.254	1.533	1.721
Cost, US $	1,493,868	531,627.2	962,240.8
ICER, US $/			
Per LY			458,390.1
Per QALY			558,973

LY, life year; QALY, quality-adjusted life year; ICER, incremental cost-effectiveness ratio; EV, enfortumab vedotin.

### Sensitivity analyses

**Figure 2:**
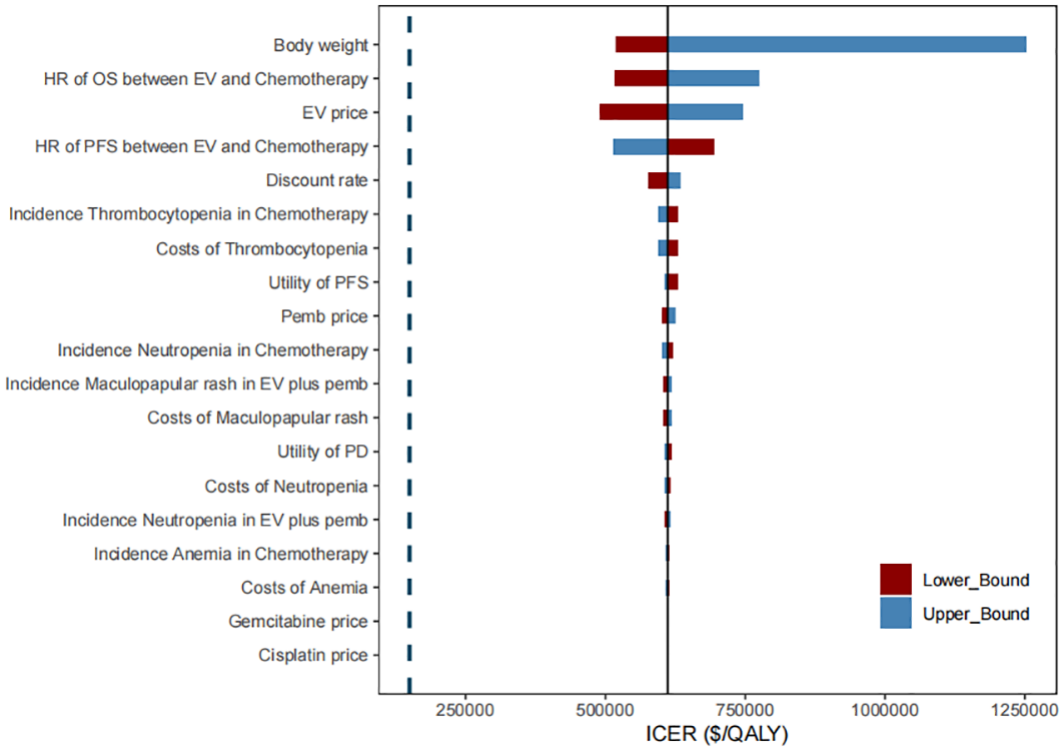
Tornado diagram of one-way sensitivity analysis of the incremental cost-effectiveness ratio (ICER) for EV plus pembrolizumab versus chemotherapy. PFS, progression-free survival; OS, overall survival; HR, hazard ratio; EV, enfortumab vedotin; pemb, pembrolizumab.

**Figure 2** presents the results of one-way sensitivity analysis. Several key variables were identified as having a significant impact on the ICER, including body weight, unit cost of EV, HR for PFS and OS, and discount rate. Despite considerable variation in these parameters, the ICER for the combination therapy of EV with pembrolizumab consistently exceeded the WTP threshold of $150,000 per QALY.

**Figure 3:**
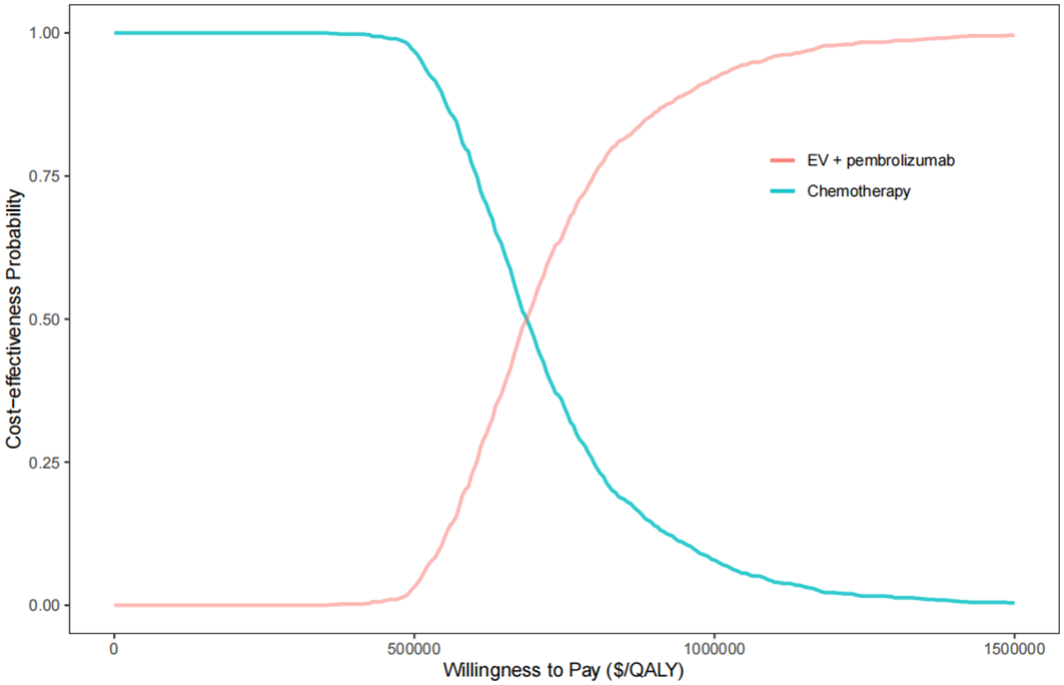
The cost-effectiveness acceptability curve for EV plus pembrolizumab versus chemotherapy. QALY, quality-adjusted life year.

The results of probabilistic sensitivity analysis, depicted in **Figure 3**, indicate that at a WTP threshold of $150,000 per QALY, the likelihood of the combination therapy of EV with pembrolizumab being cost-effective compared to chemotherapy was 0%. However, if the WTP threshold were increased to approximately $820,000 per QALY, there would be an 80% chance of being cost-effective for this combination therapy. The results of the two-way sensitivity analysis indicated that, across all utility combinations, the ICER exceeded the WTP threshold of $150,000 per QALY (**Supplementary Figure 1**).

Reducing the unit price of EV to $20 per milligram would result in a 50% probability of cost-effectiveness compared to chemotherapy at a WTP threshold of $150,000. Furthermore, reducing the unit price of EV to $15 per milligram would increase the probability of cost-effectiveness to 75% at the same WTP threshold (**Supplementary Table 2**). Additionally, if the unit prices of both EV and pembrolizumab were simultaneously reduced by 80%, there would be a 75% probability of cost-effectiveness at the specified WTP threshold.

Subgroup analyses showed that the ICER for EV in combination with pembrolizumab ranged from $563,128.5 per QALY in platinum-eligible patients to $536135.5 per QALY in platinum-ineligible patients. However, the difference in ICER values between the high and low PD-L1 expression subgroups was negligible (**Supplementary Table 3**).

## Discussion

To our knowledge, this study is the first cost-effectiveness analysis comparing EV in combination with pembrolizumab to chemotherapy regimens as first-line treatment for mUC. Based on the current study model, the ICER for EV in combination with pembrolizumab compared to platinum-based traditional chemotherapy was estimated to be $558,973 per QALY. Probabilistic sensitivity analysis indicated that the probability of EV in combination with pembrolizumab being cost-effective at a WTP threshold of $150,000 per QALY was 0%. In one-way sensitivity analysis, patient weight, unit price of EV, OS and PFS HR, and discount rate were the most influential parameters on the results. Across the broad variations in the ranges for each parameter, the ICER for the combination therapy compared with chemotherapy remained well above the WTP threshold of $150,000 per QALY.

The utility values used in the model were derived from previously published literature rather than from the EV-302 trial, which may introduce bias into the model’s predictions. Nevertheless, the one-way sensitivity analyses demonstrated that even when utility values were varied by ±20% from their baseline estimates, the results consistently remained above the WTP threshold of $150,000 per QALY. Furthermore, the two-way sensitivity analysis indicated that the interaction between the utility values for EV plus pembrolizumab and chemotherapy during PFS did not alter the conclusion that the ICER remains well above the WTP threshold. Consequently, it can be concluded that variations in utility values are unlikely to significantly influence the model’s outcomes. Research in several cancers types has shown that immunotherapy may be more cost-effective in certain patient subgroups (33, 34). In our study, patients ineligible for cisplatin who were treated with EV in combination with pembrolizumab had a lower ICER than those who were eligible for cisplatin. However, even with the reduced ICER, the value remained above the WTP threshold of $150,000 per QALY. In addition, the difference in ICERs for EV in combination with pembrolizumab versus chemotherapy between patients with high and low PD-L1 expression was minimal. However, due to the small sample sizes in each subgroup and the exploratory nature of these analyses, these results should be interpreted with caution. Data indicates that drug prices in the United States are approximately 2.78 times higher than those in other countries (35). The high cost of the drugs may impose a substantial long-term economic burden on patients, particularly those with limited financial resources or insufficient insurance coverage. This burden could manifest in increased out-of-pocket expenses, reduced access to necessary treatments, and potentially lower adherence to prescribed therapies, all of which could adversely impact patient outcomes and quality of life. Our analysis indicates that, given current prices, combination therapy with EV and pembrolizumab is not a cost-effective strategy. However, this conclusion should not lead to the default use of the less effective chemotherapy, especially within public healthcare systems where cost and accessibility are critical concerns. The Inflation Reduction Act now authorizes Medicare to negotiate directly with pharmaceutical companies to reduce the cost of the most expensive single-source brand-name drugs (36), which is a step toward making innovative, life-saving treatments more accessible and affordable. This is particularly important for public healthcare systems that aim to provide equitable care without compromising financial sustainability. While the Centers for Medicare & Medicaid Services (CMS) may use cost-effectiveness data during the initial price negotiation phase, they may focus on studies that present summary measures such as life-years gained, rather than quality-adjusted life-year metrics (37). Our study results show that the price of the combination of EV and pembrolizumab would need to be reduced, regardless of whether the outcome measure is life years gained or quality-adjusted life years, to be a viable option for public healthcare systems. This research is intended to contribute to future discussions on the pricing of EV, with a particular emphasis on guiding public healthcare systems toward more sustainable and equitable healthcare financing. Ensuring that patients across all systems, including public ones, have access to effective and economically accessible therapies is our primary goal.

As with any other model, this study also has several limitations. First, to the best of our knowledge, the EV-302 trial is the only clinical trial that has assessed the efficacy of first-line EV plus pembrolizumab in patients with mUC. Although it is a large and well-designed trial, our model is fundamentally dependent on the validity and generalizability of the study, which means that any bias inherent in the study will inevitably affect the results of our study. Second, we did not include the additional costs of all AEs that occurred during PFS. However, we do not expect that including all AEs would change the conclusions of the study, as the cost differences associated with AEs are expected to be minimal and unlikely to affect the overall results. Third, we used short-term clinical data from the EV-302 trial to extrapolate long-term survival data. Although we assessed the goodness of fit of the parameter distributions based on AIC, there remains an inherent uncertainty regarding the long-term survival benefit. We look forward to collecting more data to improve the robustness of our model. Finally, the utility values were derived from the published literature rather than directly from the EV-302 trial, which may introduce bias and potentially affect the robustness of the model. However, to account for this variability, we conducted a series of sensitivity analyses covering a wide range of utility values.

## Conclusion

Our study suggests that from the perspective of U.S. payers, EV in combination with pembrolizumab is estimated not to be cost-effective compared with chemotherapy in the first-line setting for patients with mUC at a WTP threshold of 150,000 per QALY.

## Data Availability

The original contributions presented in the study are included in the article/**Supplementary Material**. Further inquiries can be directed to the corresponding authors.
