# Supplementary material for: Cost-effectiveness of first-line enfortumab vedotin in addition to pembrolizumab for metastatic urothelial carcinoma in the United States
Source: Front Immunol. 2024 Sep 9;15:1464092. doi: 10.3389/fimmu.2024.1464092 (PMC11416998; doi:10.3389/fimmu.2024.1464092)
Supplement: Supplementary file 1 [file DataSheet1.docx]

Table S1. The values of parametric model of Chemotherapy

| Survival Curve | Distribution | AIC | Values |
| --- | --- | --- | --- |
| OS | Exponential | 1799.680 |  |
|  | Weibull | 1778.311 |  |
|  | Log-normal | 1764.967 | "lambda" 0.05871  "gamma" 0.94341 |
|  | Log-logistic | 1767.173 |  |
|  | Gompertz | 1795.210 |  |
|  | Generalized gamma | 1766.909 |  |
|  | Spline 1 | 1767.363 |  |
|  | Spline 2 | 1769.031 |  |
|  | Spline 3 | 1770.967 |  |
|  | Mix cure exp | 1801.684 |  |
|  | Mix cure wei | 1768.173 |  |
|  | Mix cure ggamma | 1767.876 |  |
|  | Mix cure llogis | 1766.509 |  |
|  | Mix cure gomp | 1785.095 |  |
|  | Mix cure lognorm | 1766.927 |  |
| PFS | Exponential | 1907.289 |  |
|  | Weibull | 1857.610 |  |
|  | Log-normal | 1814.859 |  |
|  | Log-logistic | 1812.099 |  |
|  | Gompertz | 1900.457 |  |
|  | Generalized gamma | 1816.692 |  |
|  | Spline 1 | 1814.177 |  |
|  | Spline 2 | 1809.613 |  |
|  | Spline 3 | 1795.409 | gamma0:-4.643525  gamma1:3.347913  gamma2:1.275567  gamma3:-5.080703  gamma4:4.457489  knots  0%:-0.3704841 25%:1.0863845  50%:1.8421674  75%:2.0589492  100%: 3.1931313 |
|  | Mix cure exp | 1909.295 |  |
|  | Mix cure wei | 1818.343 |  |
|  | Mix cure ggamma | 1809.915 |  |
|  | Mix cure llogis | 1808.580 |  |
|  | Mix cure gomp | 1868.034 |  |
|  | Mix cure lognorm | 1812.374 |  |

AIC=Akaike Information Criterion;exp=Exponential;wei=weibull;ggamma=Generalized gamma distribution;llogis=Log-logistic;gomp=Gompertz;lognorm=Log-normal.

Table S2. Scenario analyses of EV

| WTP ($) threshold  Price($/mg) | 100000 | 150000 |
| --- | --- | --- |
| 26 | 1.9% | 25% |
| 20 | 11.1% | 50% |
| 15 | 24.5% | 75% |

WTP=willingness-to-pay;EV,enfortumab vedotin

Table S3. The result of subgroup analyses

| Result | Cisplatin-ineligible | Cisplatin-eligible | PD-L1 high | PD-L1 low |
| --- | --- | --- | --- | --- |
| ICER($/QALY) | 536,135.5 | 563,128.5 | 569,009.8 | 567,511.1 |

ICER=incremental cost-effectiveness ratio

Figure S1. The results of the two-way sensitivity analyses for utility values


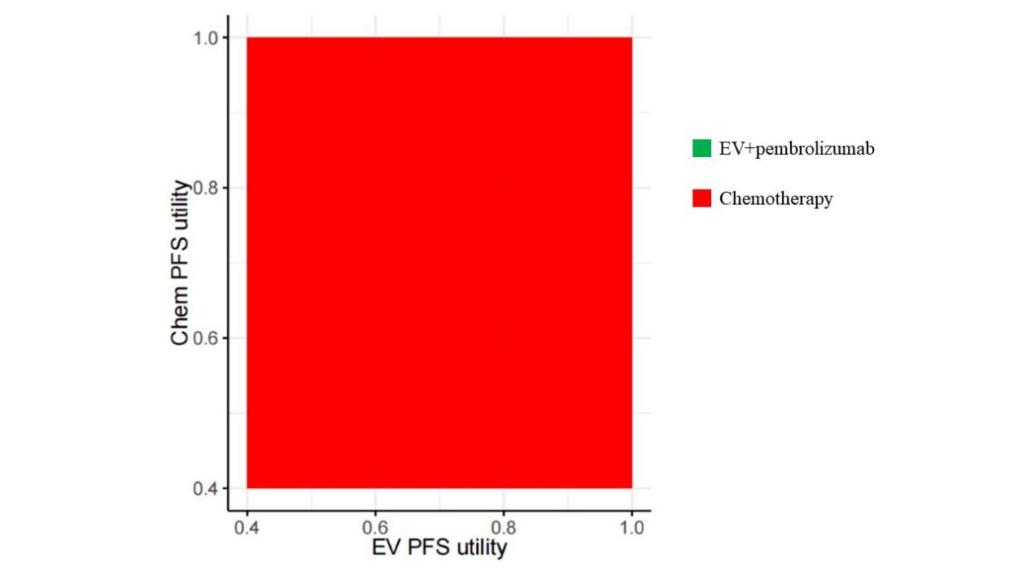


EV,enfortumab vedotin;PFS,progression-free survival.

When the utility value varies within the range, the ICER value is always higher than $150,000/QALY. This means that EV plus pembrolizumab always has no cost-effectiveness, regardless of how utility values change.
